# L1CAM promotes ovarian cancer stemness and tumor initiation via FGFR1/SRC/STAT3 signaling

**DOI:** 10.1186/s13046-021-02117-z

**Published:** 2021-10-13

**Authors:** Marco Giordano, Alessandra Decio, Chiara Battistini, Micol Baronio, Fabrizio Bianchi, Alessandra Villa, Giovanni Bertalot, Stefano Freddi, Michela Lupia, Maria Giovanna Jodice, Paolo Ubezio, Nicoletta Colombo, Raffaella Giavazzi, Ugo Cavallaro

**Affiliations:** 1 Unit of Gynaecological Oncology Research, European Institute of Oncology IRCSS, Milan, Italy; 2 Laboratory of Tumor Metastasis Therapeutics, Mario Negri Institute for Pharmacological Research – IRCCS, Milan, Italy; 3 Cancer Biomarkers Unit, Fondazione IRCCS Casa Sollievo della Sofferenza, 71013 San Giovanni Rotondo, FG Italy; 4 Present Address: Philochem AG, Otelfingen, Switzerland; 5 Department of Experimental Oncology, European Institute of Oncology IRCSS, Milan, Italy; 6 Present Address: Division of Anatomical Pathology, Santa Chiara Hospital, Trento, Italy; 7 Division of Gynecologic Oncology, European Institute of Oncology IRCSS, Milan, Italy; 8 University of Milan-Bicocca, Milan, Italy

**Keywords:** L1CAM, Ovarian cancer, Cancer stem cells, FGFR1, STAT3, Tumor initiation, Chemoresistance

## Abstract

**Background:**

Cancer stem cells (CSC) have been implicated in tumor progression. In ovarian carcinoma (OC), CSC drive tumor formation, dissemination and recurrence, as well as drug resistance, thus contributing to the high death-to-incidence ratio of this disease. However, the molecular basis of such a pathogenic role of ovarian CSC (OCSC) has been elucidated only to a limited extent. In this context, the functional contribution of the L1 cell adhesion molecule (L1CAM) to OC stemness remains elusive.

**Methods:**

The expression of L1CAM was investigated in patient-derived OCSC. The genetic manipulation of L1CAM in OC cells provided gain and loss-of-function models that were then employed in cell biological assays as well as in vivo tumorigenesis experiments to assess the role of L1CAM in OC cell stemness and in OCSC-driven tumor initiation. We applied antibody-mediated neutralization to investigate L1CAM druggability. Biochemical approaches were then combined with functional in vitro assays to study the molecular mechanisms underlying the functional role of L1CAM in OCSC.

**Results:**

We report that L1CAM is upregulated in patient-derived OCSC. Functional studies showed that L1CAM promotes several stemness-related properties in OC cells, including sphere formation, tumor initiation and chemoresistance. These activities were repressed by an L1CAM-neutralizing antibody, pointing to L1CAM as a druggable target. Mechanistically, L1CAM interacted with and activated fibroblast growth factor receptor-1 (FGFR1), which in turn induced the SRC-mediated activation of STAT3. The inhibition of STAT3 prevented L1CAM-dependent OC stemness and tumor initiation.

**Conclusions:**

Our study implicate L1CAM in the tumorigenic function of OCSC and point to the L1CAM/FGFR1/SRC/STAT3 signaling pathway as a novel driver of OC stemness. We also provide evidence that targeting this pathway can contribute to OC eradication.

**Supplementary Information:**

The online version contains supplementary material available at 10.1186/s13046-021-02117-z.

## Background

Ovarian cancer (OC) is the eighth cause of cancer but the most lethal neoplasm among female cancers worldwide, with an estimation of 295,000 diagnoses and 185,000 deaths in 2018 and a 5-year survival below 40% [8]. Many factors contribute to the high death-to-incidence ratio of OC. First, the lack of screening protocols which would allow prevention or early diagnosis. Second, the lack of specific symptoms associated to the early phases of the disease, which results in the diagnosis being done at late stages in nearly 70% of cases [23]. Third, most patients experience tumor relapse within three years from surgery and chemotherapy, which certainly represents the toughest challenge from the therapeutic standpoint. Surgical cytoreduction followed by platinum-based chemotherapy as standard first-line treatment is not sufficient to reach cure in the vast majority of the cases. Indeed, even after optimal debulking of the primary tumor and despite a high percentage of patients’ response to adjuvant chemotherapy, the rate of OC recurrence is about 70% [23]. Finally, unlike the primary tumor, recurrent OC often develops chemoresistance and becomes unresponsive to standard treatments [37].

While this has been intensely debated in the scientific community, it is now widely accepted that tumor metastasis, relapse and acquired chemoresistance are accounted for, at least to a certain extent, by a subset of cells, namely cancer stem cells (CSC, also defined as tumor-initiating cells), which fuel tumor relapse by escaping conventional therapies and initiating secondary lesions [52]. As for other malignancies, ovarian cancer stem cells (OCSC) are able to elude treatments by means of several mechanisms such as: entering into a quiescent state, which protects them from chemotherapy that targets primarily proliferating cells; activating molecular pumps to efflux drugs outside the cell; robust DNA repair ability; metabolic reprogramming; high level of adaptation (cell plasticity) to unfavorable conditions such as inflammation and nutrient deprivation; molecular mechanisms to evade the antitumor immune response; resistance to apoptotic stimuli [18, 44]. Thus, OCSC represent an optimal therapeutic target to tackle ovarian cancer. However, given the limited knowledge of their biology and pathophysiology, effective therapeutic strategies that selectively target OCSC still remain an unmet need [37].

L1 cells adhesion molecule (L1CAM, also known as L1 or CD171) is a single-passed transmembrane protein consisting, from the NH2- to the COOH-terminus, of three different portions: an extracellular domain, a transmembrane domain and a highly conserved cytoplasmic domain [43]. The ectodomain comprises six N-terminal Ig-like motifs followed by five fibronectin type III repeats. All these elements contributes to L1CAM homophilic and heterophilic interactions that impacts on a variety of signal transduction pathways [22]. Despite its initial discovery in the nervous system where it acts as a driver of neural development and plasticity [41], L1CAM is also aberrantly expressed in several solid tumors and was often associated with unfavorable prognosis in cancer patients [22]. Accordingly, the molecule is causally involved in malignancy-related processes such as invasion and metastasis [3, 29]. In OC, L1CAM has been shown to sustain tumor aggressiveness by enhancing cell proliferation, invasion and resistance to apoptosis [53, 57], and is required for intra-peritoneal tumor growth and dissemination of OC cells [4]. Finally, a causal link has been reported between L1CAM activity and OC chemoresistance [48, 53], and L1CAM-targeted treatments improve the response to chemotherapeutics [49].

However, very little and scattered information is available on the expression and function of L1CAM in CSC, although its contribution to cellular processes that are related to cancer stemness makes it a potential player in the pathophysiology of this cell subpopulation [22]. Here we report the expression of L1CAM in OCSC and unveil its novel role in specific OCSC properties. In particular, L1CAM is both sufficient and necessary for OC initiation, self-renewal and chemoresistance. Our mechanistic studies also define a novel L1CAM/FGFR1/SRC/STAT3 signaling axis which emerges as a druggable target for the OCSC eradication.

## Methods

### Cell culture

All the cells used in the study, either established cell lines or primary cell cultures, were detached using EDTA-trypsin (0.05%) and maintained at 37 °C in a humidified incubator with 5% CO_2_ tension.

Human ovarian carcinoma cell line OVCAR3 was purchased from American Type Culture Collection (ATCC) and maintained in RPMI 1640 medium (catalog no. BE12-702F; Lonza) containing 20% fetal bovine serum (FBS), 2 mM L-glutamine, 100 U/ml penicillin, 100 μg/ml streptomycin, 10 μg/ml bovine insulin. The human ovarian carcinoma cell line Ov90 was kindly provided by A. Funaro (Turin, Italy) and maintained in a 1:1 mixture of MCDB 131 (catalog no. 10372019; Thermo Fisher Scientific) and M199 (catalog no. M4530; Merck) supplemented with 15% FBS, 2 mM L-glutamine, 100 U/ml penicillin, 100 μg/ml streptomycin. The human ovarian carcinoma cell line SKOV-3 was purchased from ATCC and maintained in McCoy’s 5A medium (catalog no. 16600082; Thermo Fisher Scientific) containing 10% FBS, 2 mM L-glutamine, 100 U/ml penicillin, 100 μg/ml streptomycin. The human embryonic kidney cell line HEK293T was purchased from ATCC and cultivated in DMEM containing 10% FBS, 2 mM L-glutamine, 100 U/ml penicillin, 100 μg/ml streptomycin. Cell lines were routinely tested for mycoplasma with a PCR-based method. Cell line authentication was performed with the GenePrint 10 System (catalog no. B9510; Promega).

Fresh tissue samples were obtained upon informed consent from patients diagnosed with high-grade serous ovarian cancer undergoing surgery at the Gynecology Division of the European Institute of Oncology (Milan). Sample collection was performed under the protocol n. R789-IEO approved by the Ethics Committee of the European Institute of Oncology. All patients enrolled in this study had received no chemotherapy at the time of surgery. Tumor histology was confirmed by trained pathologists at IEO. Tissue processing and cell culture conditions have been described previously (Francavilla et al., 2017). Tumor-derived cells were used within the second passage. The purity of primary cell cultures, monitored by immunostaining for cytokeratins 5, 7, and 8, or for pan-cytokeratins, was consistently over 95%.

Ovarian cancer stem cells (OCSC) cultures were established from single-cell suspension derived from a monolayer of adherent cells after tripsynization. OCSC-enriched cultures were maintained as floating monoclonal spheres in the following medium: 1:1 mixture of DMEM:F-12, supplemented with 2% B27-supplement (catalog no. 17504044; Thermo Fisher Scientific), 2 mM L-glutamine, 100 U/ml penicillin, 100 μg/ml streptomycin, 20 ng/mL EGF, and 10 ng/mL fibroblast growth factor-2 (FGF2) and represent the first sphere generation (F1). Cells were seeded at a density of 1000 cell/ml (OVCAR3) or 2500 cells/ml (Ov90) on poly-2-hydroxyethyl methacrylate (poly-HEMA; P3932-25G, Sigma-Aldrich)-coated plates. Where indicated, spheres were propagated through a second and third generation by dissociating first-generation spheres with StemPro™ Accutase™ (catalog no. A1110501; Thermo Fisher Scientific), according to the manufacturer’s protocol, and re-seeding single-cell suspensions as described above.

Primary OCSC cultures were established from adherent (bulk) cultures as described above, seeded on poly-HEMA-coated dishes and cultured at 5000 cells/ml in serum-free MEBM™ (catalog no. CC-3151; Lonza) supplemented with 2 mM L-glutamine, 100 U/mL penicillin, 100 μg/mL streptomycin, 5 μg/mL insulin, 0.5 μg/mL hydrocortisone, 1 U/mL heparin, 2% B27, 20 ng/mL EGF, and 20 ng/mL FGF2.

### Sphere-forming assay and extreme limiting dilution analysis (ELDA)

Single-cell suspensions from either cell lines or primary cells were cultured in 6-well plates applying the conditions described above. Sphere formation was assessed after 7–8 days for cell lines and 10–14 days for primary cells. The sphere forming efficiency (SFE) was defined as the ratio between the number of spheres counted in the culture and the number of cells seeded as previously described [38].

ELDA was performed in 96-well plates with serial dilutions starting from 2000 cells/well up to 0.01 cells/well. The experiment was performed in triplicate and the wells with spheres were counted. The stem cells frequency was calculated using the ELDA software [26] available at http://bioinf.wehi.edu.au/software/elda. The input data consisted of the number of cells in each well (dose), number of wells tested (tested), number of positive wells (response), label for the population group to which cells belonged (group).

### Cell transfection

To restore L1CAM expression, OVCAR3 cells were transiently transfected with either the empty pcDNA3 vector or pcDNA3 containing the human L1CAM cDNA using the Lipofectamine™ 2000 transfection reagent (catalog no. 11668030; Thermo Fisher Scientific), according to the manufacturer’s protocol.

### Lentivirus production and cell transduction

HEK293T served as packaging cell line for lentiviral particles production using the calcium phosphate precipitation method. Ten μg of lentiviral expression plasmids were co-transfected with the following packaging plasmids and relative amount: 3 μg *PMD2G*, 5 μg *Rre* and 2.5 μg *REV*. The supernatant from HEK293T was then used to transduce the target cells (OVCAR3, OV90) adding 8 mg/mL of polybrene as adjuvant agent to increase transduction efficiency. OVCAR3 were transduced with lentiviral vectors containing either a scramble shRNA (Catalog no. CSHCTR001-LVRU6P) or the short-hairpin RNA sequences SH1 (Catalog no. HSH010390–1-LVRU6P; ggatggtgtccacttcaaa), SH3 (Catalog no. HSH010390–3-LVRU6P; ccaccaacagcatgattga). The OVCAR3-Scramble, OVCAR3-SH1 and OVCAR3-SH3 cell lines were then generated upon selection with 2 μg/mL puromycin. Ov90 were transfected with lentiviral vectors either empty (catalog no. EX-EGFP-Lv152; GeneCopoeia) or containing the cDNA for human L1CAM (catalog no. EX-Z2881-Lv152; GeneCopoeia), generating the Ov90-Mock and Ov90-L1CAM cell lines upon selection with 600 μg/ml hygromycin.

### FACS analysis and sorting

Transduced cell lines were trypsinized, counted and resuspended in FACS buffer (PSB 1x, 10% FBS). Cells were left in FACS buffer for at least 20 min as blocking step. Single-cell suspension was stained with 1 μg/ml of a monoclonal mouse anti-L1CAM antibody (clone CE7) conjugated with AF647 for 45 min at + 4 °C followed by two washes with FACS buffer. Clone CE7 was kindly provided by K. Blaser (Davos, Switzerland) and was conjugated with AF647 by the Biochemistry Facility at Cogentech (Milan, Italy). The isotype-matched antibody anti-HA (clone 12CA5) was used as a control.

For L1CAM rescuing purposes, L1CAM-positive or negative cells were sorted from OVCAR3-Scramble or OVCAR3-SH1 cells, respectively. The sorting procedure was the same as for analysis excepted for FACS buffer composition (PSB 1x, 10% FBS, 2 mM EDTA). Sorted cells were left to recover for 24 h and then were transfected with either L1CAM or empty vectors using Lipofectamine 2000 as described above. Forty-eight hours after transfection, L1CAM-positive or -negative cells were sorted from each condition. L1CAM-rescued cells together with controls were cultured as spheres and the sphere forming assay was performed as described below 7 days after sorting.

The same procedure was employed for sorting primary cells, except for the FACS buffer composition (PSB 1x, 1% FBS, 2 mM EDTA).

FACS was performed with a BD Influx Sorter (BD Biosciences).

### Cell lysates production and immunoblotting

Total proteins from both bulk and OCSC were obtained as previously described [40]. Briefly, cell pellets were boiled in lysis buffer (4% SDS, 16% glycerol, 40 mM Tris-HCl [pH 6.8]), incubated for 15 min at 90 °C, centrifuged for 5 min at 14,000 *g* and the supernatant was collected.

Nuclear and cytosolic fraction were isolated with NE-PER™ Nuclear and Cytoplasmic Extraction Reagents (catalog no. 78833, Thermo Fisher Scientific) according to the manufacturer’s protocol. Protein concentration was determined using the Pierce™ BCA Protein Assay Kit (catalog no. 23227; Thermo Fisher Scientific) according to the manufacturer’s instructions. Equal amount of proteins (20 μg) was separated by SDS-PAGE and transferred onto nitrocellulose membranes. Immunoblotting was performed with the following antibodies: L1CAM (clone UJ127; catalog no. sc-533,386; Santa Cruz Biotechnology; 1:200), vinculin (clone hVIN-1; catalog no. V9131; Sigma-Aldrich; 1:10,000), p44/42 MAPK (Erk1/2; catalog no. CST-9102; Cell Signaling technology; 1:2000), STAT3 (clone 124H6, catalog no. CST-9139; Cell Signaling technology, 1:1000), pSTAT3 (catalog no. CST-9145; Cell Signaling technology, 1:1000), α-tubulin (clone B-5-1-2; catalog no. T5168, Sigma-Aldrich; 1:15,000), HDAC1 (catalog no. ab7028; Abcam; 1:16,000), SRC (clone L4A1; catalog no. CST-2110; Cell Signaling technology; 1:1000), pSRC (catalog no. CST-2101; Cell Signaling technology; 1:1000), FGFR1 (clone M2F12; catalog no. sc-57,132; Santa Cruz biotechnology; 1:200), pFGFR1 (catalog no. CST-3471; Cell Signaling technology; 1:1000). The signal was detected by the Clarity Western ECL Substrate (Bio-Rad) and the images were acquired using ChemiDoc (Bio-Rad) and analyzed with the Fiji software.

### Immunofluorescence

OC cells were grown on coverslips and then fixed with 4% PFA for 10 min at room temperature. Where indicated, cells were permeabilized for intracellular staining by incubating coverslips in ice-cold PBS, 0.5% Triton X-100 for 3 min at 4 °C. Coverslips were then incubated for 1 h at room temperature in a humid chamber with blocking solution (PBS, 2% BSA, 5% donkey serum, and 0.05% Triton X-100). Samples were then incubated for 2 h with primary antibodies. Immunostaining was performed with the following antibodies diluted in blocking buffer: L1CAM (clone UJ127, catalog no. sc-533,386, Santa Cruz Biotechnology, 1:20 or clone 5G3, catalog no. sc-33,686, Santa Cruz Biotechnology, 1:20), STAT3 (clone 124H6, catalog no. CST-9139; Cell Signaling technology, 1:100), pSTAT3 (catalog no. CST-9145; Cell Signaling technology, 1:100). Coverslips were then incubated with Cy3-conjugated donkey anti-mouse (1:600, Listarfish) or AF488-conjugated donkey anti-rabbit (1:100, Listarfish) secondary antibodies for 45 min at room temperature. Finally, samples were washed in PBS and nuclei were counterstained with DAPI solution (0.2 μg/ml, Sigma-Aldrich). Images were acquired using the Olympus Biosystems Microscope BX71, equipped with the F-View II camera (Olympus) and the analySIS software (Soft Imaging System GmbH). For quantification, at least five fields for each condition were counted and the average of the positive/negative cells was calculated.

### Mouse studies

All animal studies were performed following protocols approved by the fully authorized animal facility of our Institutions and by the Italian Ministry of Health (as required by the Italian Law) (protocols no. 945/2016-PR and 325/2016) and in accordance with EU directive 2010/63. Mice were maintained under specific-pathogen-free conditions, housed in isolated vented cages and handled using aseptic procedures.

Mouse experiments were done in 7-week-old female NOD-SCID IL2Rgamma^null^ (NSG) strain or the athymic nude-*Foxn1*^*nu*^ mice (Charles River and Envigo Laboratories, respectively). Each experimental group included 4–5 mice. Single cell suspensions from SKOV-3 or Ov90 cell lines were mixed 1:1 with growth factor-reduced Matrigel® (catalog no. 356231; Corning) and sterile PBS (100 μl final volume). For tumor initiation assays, cells were injected subcutaneously into the mouse flank (500 cells/site for SKOV-3 and 250 cells/site for Ov90). Tumor latency was determined as the time from the injection to the formation of palpable masses. Tumor take was defined as percentage of mice with palpable tumor. For limiting dilution experiments, mice were injected with serial dilutions of Ov90 cells ranging between 25 and 25,000 cells/site. Tumor size was measured by caliper measurement and the growth curves of different tumors were calculated with the formula: Tumor volume = 1/2(length*width^2^). The stem cells frequency was calculated using the ELDA software [26] available at http://bioinf.wehi.edu.au/software/elda. The input data, as required by the software, consisted of the number of cells/mice (dose), number of mice (tested), number of mice with tumors (response), the population group to which mice belonged (group). For drug treatments, mice were injected with 25,000 Ov90 cells/site. Three days after transplantation, mice were randomized and treated with either Napabucasin (catalog no. V1386; InvivoChem) or vehicle (5 mice/group). Napabucasin was dissolved in 5% DMSO in corn oil and injected intraperitoneally at the dose of 20 mg/kg twice a week for 15 administrations.

### IC50 determination

The IC50 of Napabucasin or paclitaxel was determined by seeding Ov90 cells at 10,000 cells/well in 96-well plates in quadruplicate and treating cells for 5 days with increasing concentrations of the drugs with 1:3 dilutions. Cell death was determined 3 h after the addition of Cell Counting Kit-8 (catalog no. 96992; Sigma-Aldrich) according to the manufacturer’s protocol using microplate spectrophotometer (EPOC) automatic reader.

### Cell treatments and stimulations

All OCSC treatment experiments were performed on either transduced cell lines or primary cells. The former was seeded at 2500 cells/ml while the latter were seeded at 500 cells/ml in triplicate in poly-HEMA-coated 6-well plates. Sphere culture and SFE measurement were performed as described above.

Paclitaxel treatment (catalog no. T1912; Sigma-Aldrich) was performed with either 3, 6, 12 nM paclitaxel or vehicle. SFE was determined 7 days after treatment.

L1CAM targeting was achieved via the monoclonal antibody CE7. Cells were treated with 10 μg/ml CE7 or isotype-matched anti-HA (clone 12CA5) every 72 h. SFE was determined 7 days after treatment. Antibody-mediated L1CAM targeting in patient-derived cultures was performed on L1CAM-positive samples. SFE was determined between 10 and 14 days depending on the specific patient’s sample.

Based on IC_50_ data, Napabucasin treatment was performed using either 20, 50, 100, 150 nM of Napabucasin or vehicle. SFE was determined 7 days after treatment. Combined treatment with Napabucasin (50 nM) and paclitaxel (6 nM) was administered directly to single-cell suspension and SFE assayed after 7 days from treatment.

Apoptosis assays after drug treatments were performed using the Caspase Glo 3/7 Assay (catalog no. G8090; Promega).

The following signaling inhibitors at the indicated concentrations were used in sphere formation assays: JAK inhibitor I (CAS 457081–03-7; catalog no. 420099; Calbiochem) at 20 μM; the SRC inhibitor SU6656 (CAS 330161–87-0, catalog no. 572635; Calbiochem) at 270 nM; the FGFR1 inhibitor PD173074 (kindly provided by Pfizer) at 70 nM. SFE was determined after 7 days of treatment. Protein extracts were obtained from pre-formed spheres treated with inhibitors for 3.5 h at 37 °C.

For FGF2 stimulation, cells were serum-starved for 72 h before treating with 20 ng/ml FGF2 for the indicated time length prior to obtaining cell lysates.

### Immunohistochemistry

Fresh tissue samples were obtained upon informed consent from high-grade serous ovarian cancer patients undergoing surgery at the Gynecology Division of the European Institute of Oncology (Milan). Immunohistochemical staining was performed as follows: 3-μm-thick sections were prepared from formalin-fixed paraffin-embedded samples and dried in a 37 °C oven overnight. The sections were placed in a Bond-RX for full Automated Immunohistochemistry (Leica Biosystems) according to the following protocol. For p-STAT3 and L1CAM antibodies a double sequential automated immunohistochemistry staining was performed, where slides were pre-treated with the Epitope Retrieval Solution 2 (pH 9) (Leica Biosystems #AR9640) at 100 °C for 20 min, then incubated with p-STAT3 (catalog no. 9145, Cell Signaling Technology) (1:200), and subsequently with the primary antibody L1CAM UJ27 (Neomarkers) (1:20), both diluted in Bond Primary Antibody Diluent (catalog no. AR9352, Leica Biosystems). Tissues were incubated for a total of 16 min with post primary and polymer respectively using Polymer Refine Red Detection Kit (catalog no. DS9390, Leica Biosystems) (Fast Red chromogen) for L1CAM antibody and Bond Polymer Refine Detection Kit (catalog no. DC9800, Leica Biosystems) (DAB chromogen) for p-STAT3. Finally, the sections were counterstained with hematoxylin for 5 min, subsequently digitalized at 40× magnification using the Aperio Scanscope XT (Leica Biosystems) and analysed using the software ImageScope (Leica Biosystems) by a trained pathologist (G.B.). At least five different fields were counted for each section.

### Gene expression profile

Total RNA was extracted using RNeasy kit (Qiagen) according to manufacturer’s protocol and was quantified using a NanoDrop 2000 system (Thermo Scientific). RNA integrity was assessed by using an Agilent 2100 Bioanalyzer (Agilent, Santa Clara, CA, USA). Preparation and hybridization of cDNA samples were performed at the Cogentech Microarray Unit (Milan, Italy; www.cogentech.it). Labeled sscDNAs were hybridized on the Affymetrix GeneChip® Human Clariom S array (Thermo Fisher Scientific) which includes more than 210,000 distinct probes representative of 21,448 annotated genes [hg19; Genome Reference Consortium Human Build 38 (GRCh38)]. Samples were hybridized overnight, washed, stained, and scanned using the Affymetrix GeneChip Hybridization Oven 640, Fluidic Station 450 and Scanner 3000 7G (Thermo Fisher Scientific) to generate the raw data files (.CEL files).

Quality control and normalization of Affymetrix. CEL files were performed using Expression Console software (Affymetrix; version: 1.4.1.46) by performing “gc-sst-rma-sketch” summarization method. Gene expression data were log2 transformed before analyses. Genes with less than 20% of expression data showing at least a 1.5 -fold change in either direction from gene’s median value were excluded from Class comparison analysis. Class comparison analysis to identify differentially regulated genes was performed using t-test with a random variance model. Hierarchical clustering and heatmaps analyses were performed using Cluster 3.0 (http://bonsai.hgc.jp/~mdehoon/software/cluster/software.htm) and Java Tree View (http://jtreeview.sourceforge.net). The uncentered correlation and centroid linkage clustering method was adopted. Ingenuity pathway analysis (IPA; QIAGEN, Hilden, Germany) was performed to identify canonical pathways enriched in L1CAM regulated genes. Significantly enriched pathways were defined as those with *p*-value less than 0.05. IPA was also used to perform Upstream Regulator analysis to identify upstream transcriptional regulators (TR) that can explain the observed gene expression changes. Briefly, for each potential TR two statistical measures, an overlap p-value and an activation z- score are computed. The overlap p-value calls potential TR based on significant overlap between the set of genes (i.e., L1CAM regulated) and known targets regulated by a TR (p-value cutoff = 0.05). The activation z-score (cutoff = 2.0) was used to infer likely activation states of TR based on comparison with a model that assigns random regulation directions. Gene Set Enrichment Analysis (GSEA) was performed using GSEA_4.1.0 version and CP (canonical pathways) gene sets representing STAT3 signalling pathways (i.e. BIOCARTA_STAT3_PATHWAY; ST_STAT3_PATHWAY; WP_REGULATORY_CIRCUITS_OF_THE_STAT3_SIGNALING_PATHWAY); 1000 random permutations of gene sets were performed to calculate false discovery rate (FDR q-value); a gene set with an FDR < 25% was considered significantly enriched (default GSEA setting).

### Survival analysis

To determine the prognostic relevance of L1CAM (Affymetrix ID: 204584_at), overall survival curves of ovarian cancer patients were built using a web plotter tool (http://kmplot.com/analysis/) and combining transcriptomic data from 13 public ovarian cancer datasets [25]. Survival plots were drawn using the Kaplan-Meier method and patients were stratified according to L1CAM expression using the best cutoff values auto-selected by the plotter tool. The log-rank Mantel-Cox test was employed to determine any statistical difference between the survival curves of the cohorts.

### Statistical analysis

Independent experiments were considered as biological replicates. When performed, technical replicates deriving from the same biological replicate were averaged. For in vivo experiments, each mouse represented one biological replicate. Data are expressed as means ±SEM, calculated from at least three independent experiments. Student’s two-tailed t test was used to compare SFE values among the groups while one-way ANOVA multiple comparison test was employed to compare two or three groups and to determine statistical significance (GraphPad Prism 8). Cut-off threshold to define significance was set at *p* < 0.05. Asterisks correspond to *p*-value calculated by two-tailed, unpaired, t-test (*p < 0.05, **p < 0.01, ***p < 0.001). The sample size estimation was based on previous studies and pilot experiments.

## Results

### L1CAM is upregulated in OCSC and promotes clonogenicity and self-renewal

L1CAM is expressed at higher level in OC in respect to normal ovary (Supplementary Fig. S1A and [57]). Higher L1CAM expression has been associated to OC aggressiveness and worse prognosis [7, 19, 57], and this occurs even in Stage I/II OC patients (Supplementary Fig. S1B) who should benefit from a relatively favorable prognosis.

**Fig. 1:**
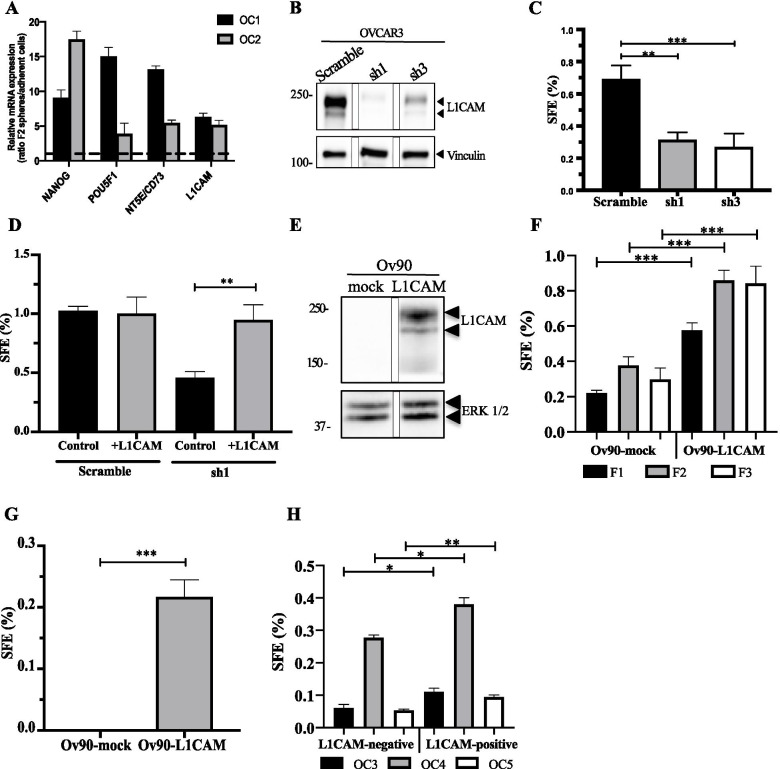
Expression and genetic manipulation of L1CAM in OCSC. **(A)** qRT-PCR for L1CAM and the stemness-related genes NANOG, POU5F1 and NT5E on second-generation spheres from OC primary cells. Data are expressed as relative mRNA level (2^-ΔΔCt^) and normalized to the corresponding adherent cultures (dashed line). Data refer to two independent primary OC samples (OC1 and OC2). **(B)** Immunoblotting on L1CAM-silenced OVCAR3 cells. L1CAM silencing was efficiently achieved through two different shRNAs. Vinculin served as loading control. The panel shows a single blot, intervening lanes were removed for clarity reasons. **(C)** Sphere forming efficiency (SFE) on L1CAM-knockdown and control OVCAR3 cells. **(D)** Sphere formation assay conducted on L1CAM-knockdown or control OVCAR3 cells upon rescuing L1CAM expression. **(E)** Immunoblot for L1CAM in L1CAM- and mock-transduced Ov90 cells. ERK1/2 served as loading control. The panel shows a single blot, intervening lanes were removed for clarity reasons. **(F)** L1CAM-trasduced Ov90cells were subjected to sphere formation assay for three consecutive sphere generations. **(G)** Sphere formation assay conducted in medium depleted of both EGF and FGF2. **(H)** Three different primary ovarian cancer samples (OC3, OC4 and OC5) were sorted according to L1CAM expression (see Supplementary Fig. S4) and subjected to sphere formation assay. For each analysis, data are expressed as means ± SEM from three independent experiments. Comparisons between experimental groups were done with two-sided Student’s t-tests; *p < 0.05, **p < 0.01, ***p < 0.001

Several studies have implicated L1CAM in malignancy-related traits of OC, including epithelial-mesenchymal transition, tumor growth and dissemination, and chemoresistance [4, 29, 30, 48, 53]. Given that ovarian cancer stem cells (OCSC) are viewed as main players in these events [37], we asked whether L1CAM plays a role in OC stemness and in OCSC-driven tumor progression. As an experimental model to investigate OCSC, we relied on the sphere culture technology that is widely used in CSC research [16, 27]. We have previously shown that culturing primary OC cells under non-adherent conditions results in the formation of spheroids highly enriched in tumor cells with stem-like properties [38]. Thus, we undertook an analogous approach to study the role of L1CAM in patient-derived OCSC. As shown in Fig. 1A, L1CAM was expressed at higher level in primary HGSOC-derived sphere-forming cells than their parental cell populations, similar to other OCSC markers including CD73 that we recently identified as an OCSC-associated gene [38].

To determine if L1CAM is required for OCSC sphere formation, OVCAR3 cells, that express high levels of L1CAM, were stably transduced with lentiviral vectors carrying two different L1CAM shRNAs which induced efficient knockdown of L1CAM (Fig. 1B and Supplementary Fig. S2A). This resulted in a dramatic reduction of sphere-forming efficiency (SFE) compared to cells transduced with a control shRNA (Fig. 1C). To rule out any off-target effect of the shRNA, we rescued L1CAM expression in L1CAM-knockdown OVCAR3 cells via transient transfection followed by FACS isolation of L1CAM-expressing cells (Supplementary Fig. S2B). This was sufficient to restore sphere formation (Fig. 1D). Furthermore, extreme limiting dilution analysis (ELDA), an assay that determines the stem cell content in a cell population [26], revealed that L1CAM-silenced OVCAR3 cells had an eight-fold lower stem cell frequency than control cells (Supplementary Fig. S2C). This set of data indicated that L1CAM is required for stemness-related properties of OC cells and for OCSC maintenance. To test whether L1CAM is also sufficient to confer a stem-like phenotype to OC cells, we applied a gain-of-function approach. L1CAM stable expression and surface exposure were achieved in the L1CAM-negative cell line Ov90 (Fig. 1E and Supplementary Fig. S3A). The ectopic expression of L1CAM led to a marked increase in Ov90 sphere formation with respect to mock-transduced cells (Fig. 1F). Notably, the positive effect of L1CAM was retained also across second (F2) and third-generation (F3) spheres (Fig. 1F), further implicating L1CAM in the expansion and maintenance of the OCSC compartment. Accordingly, the ELDA estimated a three-fold higher stem cells frequency in Ov90-L1CAM respect to Ov90-mock (Supplementary Fig. S3B). In different stem cell culture models, including OC, sphere formation requires the addition of the growth factors EGF and FGF2 [24, 38, 45]. Unexpectedly, Ov90-L1CAM cells, but not Ov90-mock, were able to generate spheres even in the absence of growth factors (Fig. 1G), pointing to L1CAM per se as a remarkable driver of OC stemness even under stringent conditions. Finally, the ectopic expression of L1CAM modulated the transcription of classical stemness-associated genes (Supplementary Fig. S3C), further supporting its role in the stem-like phenotype of OC cells.

To validate our results in an experimental model with higher clinical relevance, freshly isolated primary OC cells were sorted according to L1CAM expression (Supplementary Fig. S4) and cultured under sphere-forming conditions. L1CAM-positive cells exhibited higher SFE than their negative counterpart (Fig. 1H), consistent with a stemness-related function of L1CAM also in primary OCSC. Taken together, these results implicated L1CAM in the stem-like properties of OC cells.

### L1CAM enhances tumorigenicity of OC cells

**Fig. 2:**
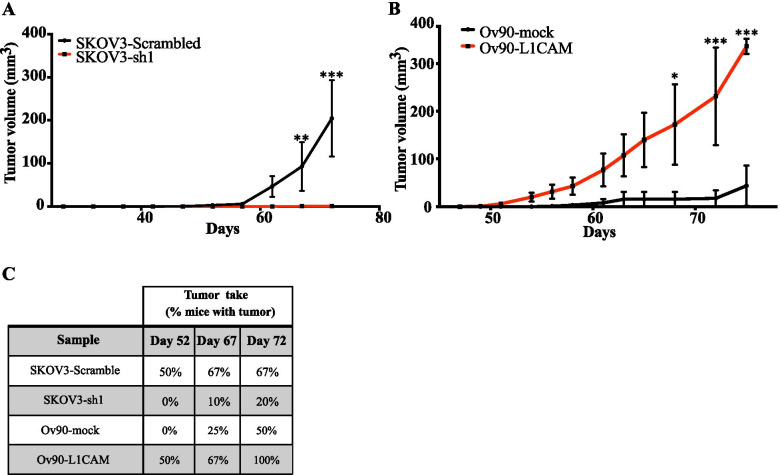
L1CAM is both necessary and sufficient for ovarian cancer tumorigenicity. **(A)** NSG mice were injected subcutaneously with a low number of either L1CAM-knockdown or control SKOV-3 cells (500 cells/site; *n* = 10) and tumor growth was monitored. **(B)** NSG mice were injected subcutaneously with a low number of either L1CAM-transduced or control Ov90 cells (250 cells/site; *n* = 8). **(C)** Tumor take is expressed as the percentage of mice with tumor at the three time points indicated. Data refer to the mice shown in (A) and (B) and are expressed as means ± SEM. Comparisons between experimental groups were done with two-way ANOVA with multiple comparison; *p < 0.05, **p < 0.01, ***p < 0.001

Tumor initiation is a defining property of CSC and, therefore, we determined the impact of L1CAM on the tumor-initiating potential of OC cells. To this goal, we relied on the xenotransplantation in immunodeficient mice of the two cell lines SKOV3 and Ov90 upon silencing and ectopic expression of L1CAM, respectively (Supplementary Fig. S5 and Fig. 1E). For L1CAM knockdown, SKOV3 were preferred to OVCAR3 because the latter are tumorigenic only when injected at high number (our unpublished data) and, therefore, are not suitable for tumor initiation experiments. NOD/SCID/IL2Rgamma-null mice were inoculated subcutaneously with 250 cells/mouse and monitored for tumor development. L1CAM silencing in SKOV3 cells almost abolished tumor initiation (Fig. 2A), while the latter was significantly enhanced in L1CAM-transfected Ov90 cells (Fig. 2B). Analogous effects were observed on tumor take, which was reduced in L1CAM-knockdown cells and increased in L1CAM-overexpressing cells (Fig. 2C). To gain further insights into the role of L1CAM in OC tumor initiation, in vivo limiting dilution experiments were conducted with Ov90-mock and Ov90-L1CAM cells. L1CAM increased 4-fold the frequency of tumor initiating cells (Supplementary Fig. S3D), confirming its ability to enhance the tumorigenic potential of OC cells.

Overall, our in vivo assays pointed to a causal role of L1CAM in the tumor initiation capacity of OC cells.

### L1CAM increases OCSC chemoresistance and is a druggable target in OCSC

**Fig. 3:**
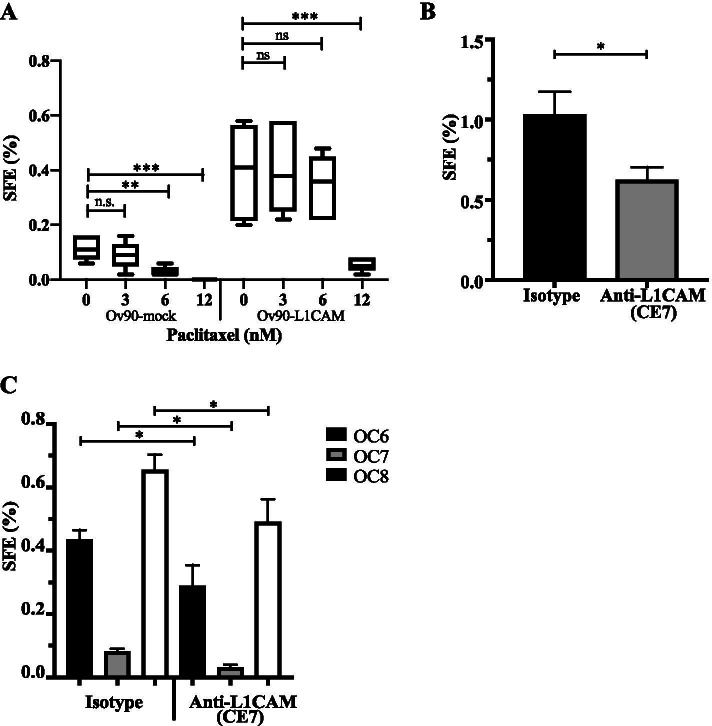
L1CAM confers chemoresistance and is a druggable target. **(A)** L1CAM-transduced Ov90 cells were treated with different doses of paclitaxel and subjected to sphere formation assay. **(B)** SFE assay in OVCAR3 cells treated with the monoclonal neutralizing antibody CE7 (20 μg/ml). **(C)** Three different primary ovarian cancer samples (OC6, OC7 and OC8) were treated with the neutralizing antibody CE7 (20 μg/ml) and subjected to an SFE assay. For each analysis, data are expressed as means ± SEM from three independent experiments. Comparisons between experimental groups were done with two-sided Student’s t-tests; *p < 0.05, ***p < 0.001; ns = not significant

OCSC-driven chemoresistance is considered a major player in OC recurrence. To assess if L1CAM is involved in determining OCSC chemoresistance, we assayed mock or L1CAM-transfected Ov90 cells for sphere formation upon treatment with increasing concentrations of paclitaxel. The drug caused a dose-dependent reduction in the SFE of Ov90-mock cells, while it exhibited a much lower toxicity toward Ov90-L1CAM cells (Fig. 3A). Indeed, 6 nM paclitaxel was sufficient to kill approximately 90% of Ov90-mock OCSC, while no significant cytoxicity was observed in Ov90-L1CAM OCSC (Fig. 3A). This pointed to L1CAM as a causal player in OCSC chemoresistance. It is noteworthy that the bulk populations (i.e., adherent cultures not enriched for OCSC) of Ov90-mock and Ov90-L1CAM cells showed a very similar response to paclitaxel (Supplementary Fig. S6A), which would imply that L1CAM-induced chemoresistance is OCSC-specific.

We then asked whether L1CAM represents a druggable target in OCSC. To address this question, we assayed for OVCAR3 sphere formation upon treatment with the L1CAM-neutralizing antibody CE7 [4, 57]. As shown in Fig. 3B, the neutralization of endogenous L1CAM caused a significant reduction in sphere formation. To validate this result in patient-derived models, three independent primary OC cultures were treated with the CE7 antibody. Sphere formation efficiency was variable among the three samples, consistent with different contents of OCSC. Notwithstanding, CE7 reduced the sphere-forming capacity in all primary cultures (Fig. 3C). We also tested whether the neutralization of endogenous L1CAM affected the response of OCSC to chemotherapy. Indeed, CE7 enhanced the cytotoxic effect of carboplatin on OCSC in both OVCAR3 and primary OC cells (Supplementary Fig. S6B and S6C).

Overall, this set of experiments suggested that L1CAM enhances OCSC resistance to chemotherapy and its targeting reduces OCSC frequency.

### L1CAM-induced STAT3 activation in OCSC

**Fig. 4:**
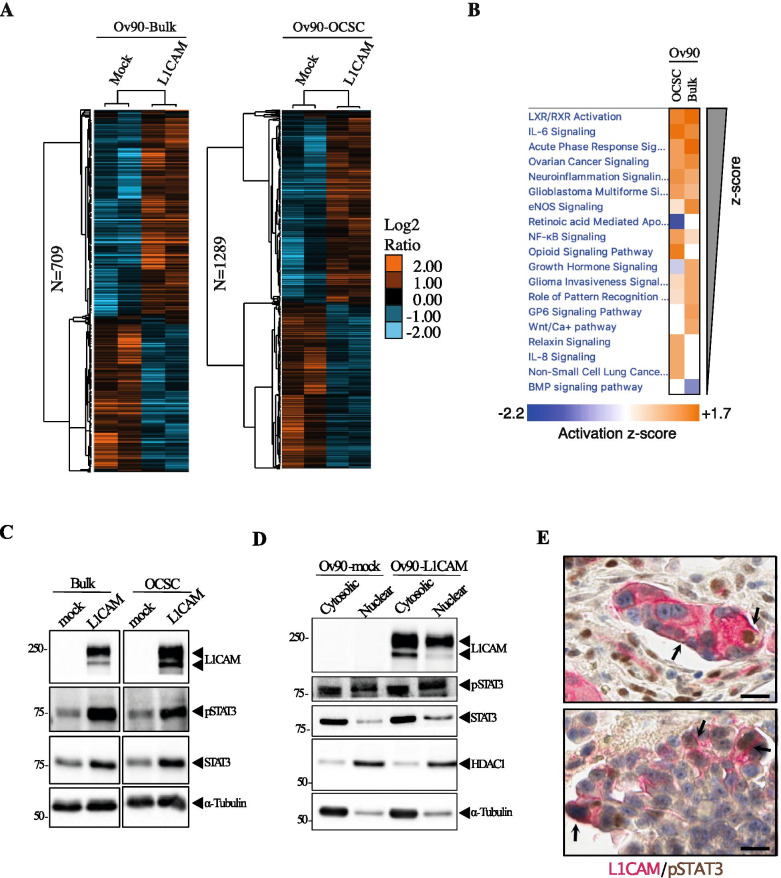
Ov90 transcriptome profiling upon L1CAM ectopic expression and validation at protein level. **(A)** Hierarchical clusters of L1CAM regulated genes in Ov90-Bulk (*n* = 709) or Ov90-OCSC experimental conditions (*n* = 1289). **(B)** IPA-upstream modulator analyses of Ov90-bulk and Ov90-OCSC overexpressing L1CAM. The heatmap represents activation z-scores (≥1; *p*-value< 0.05) of the mechanisms shown on the left which were ordered from the higher (on top) to the lower (bottom) z-score value (average of the two experimental conditions). **(C)** Immunoblotting on L1CAM-transduced and control Ov90 cells, either as bulk or OCSC. α-tubulin served as loading control. **(D)** Immunoblotting on nuclear and cytosolic fractions of serum-starved Ov90-mock and Ov90-L1CAM. HDAC1 and α-tubulin served as nuclear and cytosolic loading control, respectively. **(E)** Sections of two representative OC samples co-stained for L1CAM (red) and pSTAT3 (brown). Arrows indicate L1CAM and pSTAT3 co-localization in tumor cells. Scale bar, 20 μm

To shed light on the molecular mechanisms underlying the function of L1CAM in OCSC, we profiled the transcriptomes of Ov90-mock and Ov90-L1CAM cells either as bulk or as OCSC-enriched spheres. The ectopic expression of L1CAM resulted in the differential regulation (*p* < 0.05; Welch’s test) of 792 transcripts in adherent cells (709 unique coding genes; Supplementary Table S1) and 1462 transcripts in OCSC (1289 unique coding genes; Supplementary Table S2) (Fig. 4A), which would support a pleiotropic role of L1CAM in OC stemness. To rewire OC relevant pathways controlled by L1CAM, we performed Ingenuity Pathway Analysis (IPA) of L1CAM-regulated genes in adherent cells and in OCSC (see Methods). This revealed, among the top-ranking pathways, the activation of IL-6 signaling in L1CAM-expressing cells (Fig. 4B; Supplementary Table S3). To gain further insight into the interplay between L1CAM and IL6/STAT3 signaling, we performed a gene set enrichment analysis (GSEA) using Canonical Pathways (CP) genesets (*n* = 3; see Methods). The GSEA showed an enrichment for STAT3 pathway in Ov90-L1CAM-derived OCSC as compared to L1CAM-negative OCSC (NES = 1.5; *p*-value = 0.04; q-value = 0.086; Supplementary Fig. S7A). Consistently, total and phosphorylated STAT3 (pSTAT3) levels were increased by L1CAM in both bulk and OCSC populations (Fig. 4C). Conversely, STAT3 phosphorylation was reduced upon L1CAM silencing in OVCAR3-derived OCSC (Supplementary Fig. S7B), implying that L1CAM is both sufficient and necessary for STAT3 activation.

As a transcription factor, STAT3 activity is related to its nuclear translocation [33]. To test if L1CAM is involved in this process, cytosolic and nuclear protein fractions extracted from Ov90-mock and Ov90-L1CAM were assayed for STAT3 levels. As shown in Fig. 4D, STAT3 was more abundant in the nuclear fraction of L1CAM-expressing cells respect to the control cells. Higher nuclear translocation of STAT3 in Ov90-L1CAM cells was also confirmed by immunofluorescence staining (Supplementary Fig. S7C). Of note, we also found high accumulation of L1CAM itself in the nucleus of Ov90-L1CAM cells (Fig. 4D).

To understand whether the L1CAM/STAT3 interplay might occur also in human OC specimens, we performed immunohistochemistry co-staining for L1CAM and pSTAT3 in a small cohort (*n* = 20) of patient biopsies. This revealed that L1CAM was co-expressed with pSTAT3 in a subpopulation of cancer cells (Fig. 4E), and the frequency of double positive cells varied greatly among samples ranging from 2 to 60% of tumor cells (data not shown). The co-localization of L1CAM and pSTAT3 was also detected in several tumor vessels (Supplementary Fig. S7D), in accordance with our previous demonstration of a functional cross-talk between the two molecules in tumor endothelium [40]. Overall, these observations indicate that L1CAM stimulates STAT3 activation in OCSC and suggests that the crosstalk between L1CAM and STAT3 signaling occurs in human OC and might contribute to the clinical evolution of the disease.

### STAT3 is a druggable target in L1CAM-driven OC

**Fig. 5:**
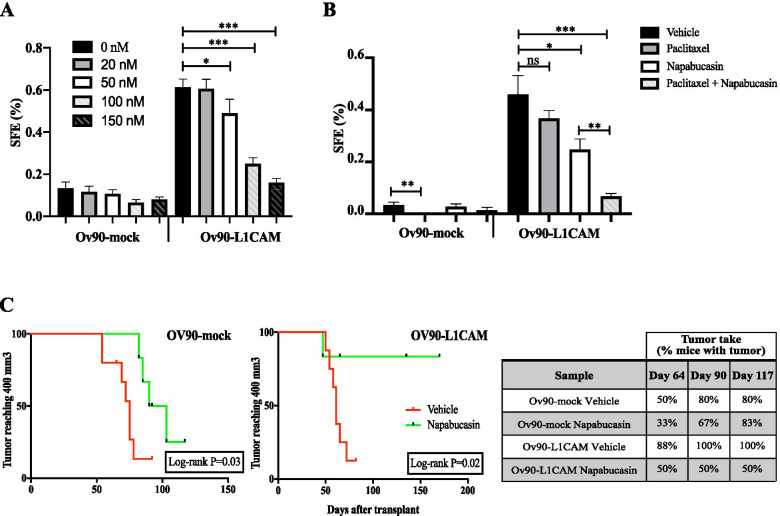
STAT3 is required for L1CAM-driven clonogenic advantage and is a suitable target both in vitro and in vivo. **(A)** Ov90-mock and Ov90-L1CAM cells were treated with the indicated concentration of Napabucasin and subjected to sphere formation assay. **(B)** SFE assay in Ov90-mock and Ov90-L1CAM cells treated with Napabucasin (50 nM) and paclitaxel (6 nM), either alone or in combination. For each analysis, data are expressed as means ± SEM from three independent experiments. Comparisons between experimental groups were done with two-sided Student’s t-tests. **(C)** Kaplan-Meier curves showing tumor volume over time in mice treated with Napabucasin upon subcutaneous injection of either Ov90-mock or Ov90-L1CAM cells. Tumor volume equal to 400 mm^3^ was defined as data censoring criterion. Comparisons between experimental groups were done with Log-rank (Mantel-Cox) test; *p < 0.05, ***p < 0.001; ns = not significant

We then asked whether the activity of STAT3 is required for L1CAM function in OCSC. To address this question, OCSC were treated with the STAT3 inhibitor Napabucasin [34]. Ov90-L1CAM sphere formation was reduced by Napabucasin in a dose-dependent manner, while no significant effect was observed in Ov90-mock cells (Fig. 5A). Given that L1CAM promoted OCSC chemoresistance (Fig. 3A), we tested whether this phenomenon involved STAT3 activity. Indeed, Napabucasin increased dramatically the response to paclitaxel of L1CAM-expressing OCSC, with sphere formation being almost abolished under conditions in which the two drugs alone had negligible effect (Fig. 5B). Accordingly, the combination of paclitaxel and Napabucasin induced apoptosis specifically in L1CAM-expressing Ov90 cells, showing a stronger effect than either drug alone (Supplementary Fig. S8). These findings suggest that both L1CAM-dependent sphere formation and OCSC chemoresistance are mediated by STAT3 signaling.

To determine if STAT3 is also required for L1CAM-induced tumor initiation, we injected immunodeficient mice with Ov90-mock and Ov90-L1CAM cells and treated them with Napabucasin starting three days after injection. The tumorigenicity of Ov90-L1CAM cells was markedly reduced upon STAT3 inhibition, with tumor initiation occurring only in 50% of the Napabucasin-treated mice as opposed to 100% in the vehicle-treated group. In contrast, Napabucasin did not affect tumor initiation in Ov90-mock-injected mice (Fig. 5C). Besides reducing tumorigenesis, STAT3 inhibition also caused a dramatic delay in the growth of Ov90-L1CAM tumors, but not of Ov90-mock (Fig. 5C). Thus, STAT3 blockade inhibited both L1CAM-dependent tumor initiation and growth.

### L1CAM stimulates STAT3 activation via SRC

**Fig. 6:**
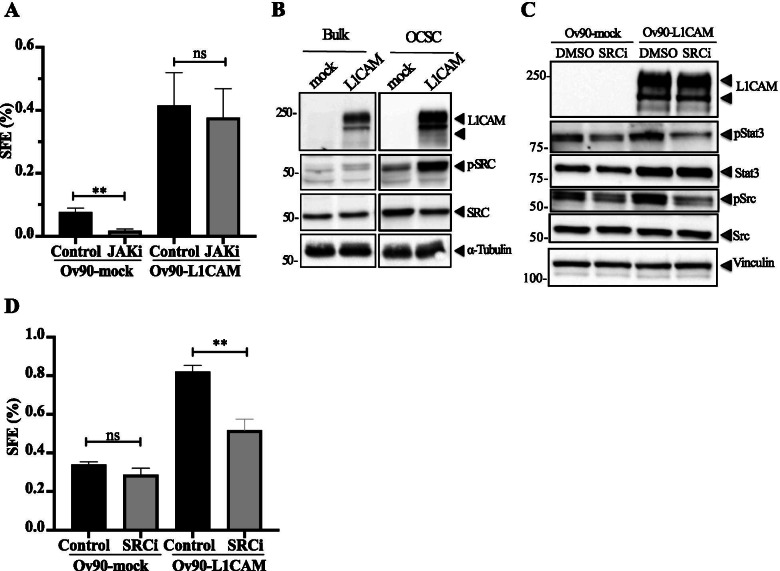
SRC is involved in L1CAM-dependent STAT3 phosphorylation. **(A)** SFE assay was conducted on Ov90-mock and Ov90-L1CAM upon treatment with 20 μM JAK inhibitor (JAKi). **(B)** Immunoblots for phosphorylated or total SRC on serum-starved Ov90-mock and Ov90-L1CAM cells. α-tubulin served as loading control. **(C)** Second-generation spheres from Ov90-mock and Ov90-L1CAM cells were lysed and immunoblotted for phospho-STAT3, total STAT3, phospho-SRC and total SRC. Vinculin served as loading control. **(D)** SFE assay on Ov90-mock and Ov90-L1CAM cells upon SU6656 (SRCi) treatment. For each analysis, data are expressed as means ± SEM from three independent experiments. Comparisons between experimental groups were done with two-sided Student’s t-tests; **p < 0.01; ns = not significant

STAT3 activation relies on the phosphorylation of the Tyr705 residue, which is commonly carried out by JAK1/2 kinases [28]. Along this line, we have previously reported that L1CAM enhances STAT3 activation in endothelial cells by inducing its JAK1/2-mediated phosphorylation [40]. Therefore, we hypothesized an L1CAM/JAK/STAT3 axis also in OCSC. Surprisingly, however, the chemical inhibition of JAK1/2 did not affect sphere formation in Ov90-L1CAM cells, while a significant decrease was observed in control cells (Fig. 6A). This implicated the JAK pathway in L1CAM-negative OCSC, while L1CAM-dependent clonogenic potential was JAK-independent. This ruled out the possibility that L1CAM induced STAT3 activation via JAK and prompted us to identify the alternative mechanism of STAT3 activation operating downstream of L1CAM. We focused on SRC, which has been identified as a non-canonical kinase for STAT3 that phosphorylates the latter at the same Tyr705 residue [50]. First, we tested whether the ectopic expression of L1CAM had any impact on SRC activation. Indeed, Ov90-L1CAM cells exhibited higher constitutive activation of SRC as compared to Ov90-mock cells, a difference that became remarkable in OCSC (Fig. 6B). Accordingly, silencing endogenous L1CAM in OVCAR3 cells markedly reduced SRC activation (Supplementary Fig. S7E). We then tested the role of SRC in L1CAM-induced activation of STAT3 in OCSC by treating the latter with the SRC inhibitor SU6656 [6]. As shown in Fig. 6C, the constitutive STAT3 phosphorylation in Ov90-L1CAM cells was efficiently inhibited by SU6656, suggesting that SRC phosphorylates STAT3 in OCSC consistent with previous findings [50]. Moreover, SU6656 reduced the sphere-forming potential of L1CAM-expressing OCSC to the level of control cells (Fig. 6D), which supported the functional relevance of L1CAM-elicited SRC activity in OC stemness. It is worth nothing that, although SRC inhibition slightly reduced STAT3 phosphorylation also in Ov90-mock cells (Fig. 6C), this had no impact on their clonogenic potential (Fig. 6D), consistent with the hypothesis that the SRC/STAT3 pathway is specifically associated with L1CAM function in OCSC.

These results indicate that L1CAM stimulates SRC-mediated STAT3 activation which enhances stemness-related properties in OCSC.

### FGFR1 is an upstream effector of L1CAM signaling in OCSC

**Fig. 7:**
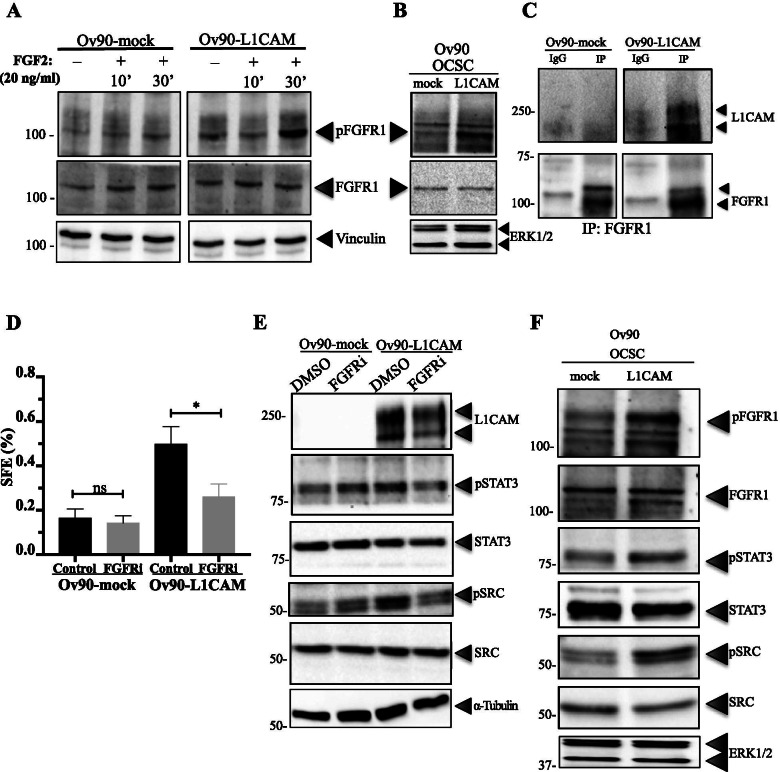
FGFR1 is an upstream effector of L1CAM signaling in OCSC. **(A)** Ov90-mock and Ov90-L1CAM cells were treated with 20 ng/ml of FGF2 for the indicated time lengths, followed by immunoblotting for phosphorylated or total FGFR1. Vinculin served as loading control. **(B)** Immunoblotting for phosphorylated or total FGFR1 was also performed on second-generation spheres from Ov90-mock and Ov90-L1CAM cells. ERK1/2 served as loading control. **(C)** Protein extracts from Ov90-mock and Ov90-L1CAM were immunoprecipitated with the anti-FGFR1 antibody (or with irrelevant IgG) and immunoblotted for L1CAM (top) and FGFR1 (bottom). **(D)** Ov90-mock and Ov90-L1CAM cells were treated with 70 nM PD173074 (FGFRi) and subjected to the SFE assay. Data are expressed as means ± SEM from three independent experiments. Comparisons between experimental groups were done with two-sided Student’s t-tests; *p < 0.05; ns = not significant. **(E)** Ov90-mock and Ov90-L1CAM cells were treated with PD173074, followed by immunoblotting for the indicated proteins. α-tubulin served as loading control. **(F)** Ov90-mock and Ov90-L1CAM cells were grown as spheres under non-permissive, growth factor-free culture conditions (see Fig. 1G) prior to immunoblotting for the indicated proteins. ERK1/2 served as loading control

Both SRC and STAT3 require tyrosine phosphorylation for their activation which, however, cannot be directly accounted for by L1CAM since the latter has no kinase activity. In an attempt to identify the molecular link between L1CAM and the SRC/STAT3 pathway, we reasoned that the immunoglobulin-like cell adhesion molecule NCAM, that is structurally and functionally related to L1CAM [10], is able to stimulate fibroblast growth factor receptor-1 (FGFR1) function resulting in SRC activation [20]. In addition, L1CAM itself has been proposed to stimulate FGFR signalling [32, 42]. On this basis, we first tested whether the ectopic expression of L1CAM in Ov90 cells affected FGFR1 activity, as determined by its phosphorylation status. Indeed, basal phosphorylation of FGFR1 was higher in Ov90-L1CAM than Ov90-mock. Moreover, the expression of L1CAM enhanced the autophosphorylation of FGFR1 in response to its canonical ligand FGF2 (Fig. 7A). L1CAM-induced activation of FGFR1 was even more pronounced in Ov90 spheres (Fig. 7B; see also Fig. 7F), suggesting its involvement in OC stemness. On these premises, we asked whether L1CAM-dependent stimulation of FGFR1 could reflect a physical interaction between the two proteins. Indeed, co-immunoprecipitation experiments in Ov90-L1CAM cells revealed that L1CAM forms a complex with FGFR1 (Fig. 7C).

To explore the functional relevance of the L1CAM/FGFR1 interplay in OCSC, cells were subjected to sphere formation assays in the presence of the FGFR inhibitor PD173074 [51]. The latter reduced significantly the sphere-forming ability of Ov90-L1CAM cells while leaving unaffected that of Ov90-mock cells (Fig. 7D).

After implicating FGFR1 as an L1CAM effector in OCSC, we checked whether the signaling cascade downstream of L1CAM/FGFR1 involved SRC and/or STAT3. To this purpose, Ov90-L1CAM and Ov90-mock OCSC were treated with PD173074 prior to assaying for SRC and STAT3 activation by immunoblotting. As shown in Fig. 7E, FGFR inhibition abolished L1CAM-dependent phosphorylation of both SRC and STAT3, thus supporting the notion that this event is mediated by FGFR1 signaling. Since L1CAM per se was sufficient to promote sphere formation even in the absence of EGF and FGF2 (Fig. 1G), we checked whether also its downstream signaling cascade was activated under such stringent conditions. Indeed, OCSC derived from Ov90-L1CAM cells, but not from Ov90-mock, exhibited constitutive activation of FGFR1 as well as of SRC and STAT3 (Fig. 7F), confirming the role of L1CAM as a cell-autonomous stemness driver even in the absence of exogenous stimuli.

Overall, these data unraveled a novel L1CAM/FGFR1/SRC/STAT3 axis that enhances stemness-related properties in OCSC.

## Discussion

We report here the novel role of L1CAM in OCSC pathophysiology via the L1CAM/FGFR1/SRC/STAT3 signaling axis. L1CAM has long been known as a key player in tumor cell migration and invasion in different cancer types including OC [29, 57]. However, only a few sporadic reports have implicated L1CAM in cancer stem cells (CSC). For example, L1CAM has been recently proposed as a biomarker of CSC in colorectal cancer [14]. Along this line, Ganesh et al. have implicated L1CAM-expressing CSC in the initiation of colorectal cancer metastasis. This study, furthermore, demonstrated that L1CAM contributes to tumor dissemination and chemoresistance as well as to CSC-driven generation of organoids [21]. L1CAM-related cancer stemness has also been reported in glioblastoma where L1CAM regulates the checkpoint-mediated DNA damage response of CSC by modulating the expression of the early checkpoint response component NSB1 [11]. With regard to OC, L1CAM has been recently proposed to mark, in association with CD133, a subpopulation of cancer cells with stem-like features [54]. That study, despite employing cell lines only and providing limited mechanistic insights, lends further support to our findings on L1CAM as a driver of OC stemness. Conversely, L1CAM seems to suppress stemness-related traits in pancreatic carcinoma [13], suggesting that the role of L1CAM in CSC depends on the tumor type and/or on the cellular context. Regardless, the molecular mechanisms that underlie L1CAM function in CSC have remained elusive. Our study provides the first evidence for a novel L1CAM/FGFR1/SRC/STAT3 signaling pathway as a key player in OCSC. While the interplay between L1CAM and FGFR1 signaling has been extensively documented in the nervous system where it enhances neurite outgrowth [15], little information is available on its possible involvement in cancer cells. To our knowledge, a functional crosstalk between L1CAM and FGFR1 has been documented only in glioblastoma where it promoted cell motility and proliferation [42]. Interestingly, indirect evidence suggested a cooperation between L1CAM and the FGFR signaling machinery also in ovarian cancer cell proliferation [57]. Yet, no evidence was provided so far on the involvement of the L1CAM/FGFR1 interplay in CSC. Our study shows that not only L1CAM interacts with and stimulates the activation of FGFR1, but also that FGFR1 activity is a prerequisite for L1CAM-induced sphere formation in OC cells, a widely used surrogate assay for stemness [27]. In this regard, we made the unexpected observation that the mere expression of L1CAM per se was sufficient to induce sphere formation in OCSC even in the absence of exogenous FGF2. The latter, in fact, is generally considered as an indispensable stimulus for sphere formation ([46], and references therein), as it is also supported by the lack of spheres in L1CAM-negative OC cells in the absence of FGF2. Our data with FGF2-deprived medium, while confirming the requirement for FGFR signaling during sphere formation, indicate that this can also be obtained via L1CAM function in a FGF2-independent manner. This further implicates the L1CAM/FGFR1 axis in OC stemness and points to L1CAM as a master regulator of OCSC even under non-permissive experimental conditions.

In this context, L1CAM expression and exogenous FGF2 appear to exert the same spherogenic function in OC cells. Nevertheless, the possibility remains that the FGFR1 signaling cascade elicited by L1CAM differs from that activated by the conventional ligand FGF2. Indeed, our previous work on neural cell adhesion molecule (NCAM), an immunoglobulin-like family member which shares many structural and functional features with L1CAM (including the ability to trigger FGFR1 activation [15];), revealed that this molecule acts as an FGFR1 ligand, yet inducing a signaling cascade and a cellular response that are divergent from those occurring downstream of FGF2 [20]. Of note, the NCAM/FGFR1 pathway, but not FGF2/FGFR1, induced sustained SRC activation, which is consistent with our finding on basal SRC activation downstream of L1CAM/FGFR1 in OCSC. Future research should address to what extent the L1CAM-triggered FGFR1 signaling cascade overlaps with that of the classical ligand FGF2 or rather there is an L1CAM-specific mode of activation of FGFR1. In the latter case, it will be interesting to clarify if such L1CAM-specific pathway is restricted to OCSC or operates also in other cellular contexts.

Our discovery of the crosstalk with the FGFR1 signaling machinery as a major determinant of L1CAM function in OCSC does not rule out the possibility that L1CAM modulates OC stemness also through additional mechanisms. For example, we have found a remarkable accumulation of L1CAM in the nucleus of Ov90-L1CAM cells. While this may be an artifact deriving from the ectopic expression of the protein, it is consistent with previous observations of nuclear translocation of L1CAM in various cell types including OC cells [11, 31, 47]. In those studies, nuclear L1CAM appeared to impact a wide range of cellular functions by regulating the expression of specific genes, most likely via interacting with DNA-binding such as transcription factors and/or with DNA itself [11, 39]. Combining these findings with our present study, it is conceivable that the nuclear accumulation of L1CAM is involved in the modulation of stemness-related genes which in turn contribute to the OCSC phenotypical and functional traits. In this case, L1CAM would exert spatially distinct roles in OCSC, activating FGFR1 at the cell surface and regulating gene transcription in the nucleus. Future research should test this hypothesis and clarify the relative contribution of the two phenomena to L1CAM-driven OC stemness.

The L1CAM/STAT3 axis is not restricted to OCSC. Indeed, we have previously reported that L1CAM stimulates STAT3 activation in endothelial cells resulting in cell proliferation and migration [40]. However, the L1CAM/STAT3 crosstalk in the endothelium relies on L1CAM inducing the classical IL-6/JAK2/STAT3 pathway via the upregulation of Interleukin-6 (IL-6) and of its receptor IL-6Rα, resulting in the activation of JAK2 and ultimately STAT3 phosphorylation [40]. In contrast, L1CAM-induced activation of STAT3 in OCSC does not involve the JAK2 pathway and is instead mediated by the FGFR1/SRC axis. Thus, L1CAM-dependent STAT3 activation occurs across different cell types, yet the underlying molecular pathways appears to be cell context-dependent.

The role of STAT3 in CSC has been described in various tumor types including OC [35]. However, to our knowledge only the canonical JAK2/STAT3 pathway has been implicated in OCSC function, including tumor initiation and chemoresistance [1, 9, 17, 55]. Our findings on the L1CAM/FGFR1/SRC/STAT3 axis, while confirming and extending the functional contribution of STAT3 to OC stemness, unveiled a novel activation cascade which includes potential targets that could be explored for OC eradication. In this context, our proof-of-concept assays, both in vitro and in mouse xenografts, revealed that STAT3-targeted therapy exhibits a markedly higher efficacy in L1CAM-expressing OC, while no or little effect was observed in L1CAM-negative cells. From a mechanistic standpoint, this observation might imply that L1 activity makes OCSC addicted to STAT3 function and, therefore, is required for STAT3 inhibitors to defeat OC stemness. Regardless the mechanism, these findings might impact the therapeutic decision-making process. Several clinical and preclinical studies with STAT3 inhibitors in cancer have led to the conclusion that, in order to improve the response to such drugs, one of the most prominent challenges is identifying patients who are likely to respond to the treatment based on predictive biomarkers [56]. It is then tempting to speculate that L1CAM expression may offer a simple and straightforward tool for the a priori selection of patients more responsive to STAT3-targeted drugs, a hypothesis that deserves further investigation. Given that both L1CAM and activated STAT3 have been implicated in different tumor types [29, 56], this paradigm might not be limited to OC.

While OC is commonly responsive to first-line chemotherapy, most patients undergo tumor relapse and the recurrent disease often becomes unresponsive to subsequent treatments. Such a phenomenon is ascribed to the emergence of cancer cell subpopulations, often identified as CSC, that are not eliminated by chemotherapeutics. Previous studies provided circumstantial evidence that L1CAM may be associated with acquired chemoresistance. First, higher levels of L1CAM have been found in drug-resistant OC as compared to responsive tumors [5, 12, 36]. Second, L1CAM was enriched upon chemotherapy in OC patients [2]. Third, the loss or inactivation of L1CAM sensitized OC cells to chemotherapeutics [48, 49]. We now report that OCSC display increased resistance to chemotherapy upon forced L1CAM expression, while the antibody-mediated neutralization of endogenous L1CAM results in higher OCSC chemosensitivity. Together with the other findings described here, these data imply that L1CAM not only orchestrates the tumor-initiating function of OCSC, but it also fuels OCSC-driven chemoresistance. An important corollary to this paradigm is that combining L1CAM-targeted treatments with conventional chemotherapy may eradicate OC by hitting the residual population of tumor-initiating and drug-resistant OCSC. Besides chemotherapy, targeting L1CAM may be beneficial also in the context of radiotherapy. Indeed, a recent study showed that silencing L1CAM in the double-positive L1CAM+/CD133+ stem-like cells overcame their radioresistance [54]. While L1CAM inactivation has already been shown to improve the OC response to cytostatic drugs, testing to what extent this involves targeting OCSC will require ad hoc preclinical models and clinical trials specifically designed to consider the role of OCSC in tumor relapse, metastasis initiation and acquired chemoresistance.

## Conclusion

We unveiled L1CAM as a master regulator of OCSC pathophysiology through a previously unrecognized L1CAM/FGFR/SRC/STAT3 signaling pathway, thus shedding light on novel molecular mechanisms that sustain the tumor-supporting function of OCSC. We believe that these findings may have relevant therapeutic implications which should be explored in appropriate preclinical models and eventually in the clinical setting.

## Supplementary Information

**Additional file 1.**

**Additional file 2.**

## Data Availability

All data generated or analyzed during this study are included in the article and/or in the Supplementary Information.

## Abbreviations

L1CAM: L1 cell adhesion molecule
OC: Ovarian cancer
CSC: Cancer stem cells
OCSC: Ovarian cancer stem cells
FGFR1: Fibroblast growth factor receptor-1
FGF2: Fibroblast growth factor-2
EGF: Epidermal growth factor
STAT3: Signal transducer and activator of transcription-3
IL-6: Interleukin-6
ELDA: Extreme limiting dilution assays
SFE: Sphere-forming efficiency
GSEA: Geneset enrichment analysis
