# Supplementary material for: L1CAM promotes ovarian cancer stemness and tumor initiation via FGFR1/SRC/STAT3 signaling
Source: J Exp Clin Cancer Res. 2021 Oct 13;40:319. doi: 10.1186/s13046-021-02117-z (PMC8513260; doi:10.1186/s13046-021-02117-z)
Supplement: Supplementary file 1 — Additional file 1. [file 13046_2021_2117_MOESM1_ESM.pdf]

Supplementary figure legends

Supplementary Figure S1. L1CAM clinical relevance in OC. (A) L1CAM is highly expressed in OC (red) with respect to normal ovary (grey). RNASeq data from http://gepia.cancer-pku.cn. **(B)** Kaplan-Meier curves show 5-year overall (left) and progression-free survival (right) in a cohort of 133 patients with Stage I/II OC. Red line, high L1CAM expression; black line, low L1CAM expression. Data were obtained from www.kmplot.com.

Supplementary Figure S2. L1CAM silencing in OVCAR3 OC cell line. (A) Representative immunofluorescence images of OVCAR3 cells silenced for L1CAM and then stained for L1CAM (red) and counterstained with DAPI (blue). The silencing of L1CAM was efficiently achieved with two different shRNAs. Scale bars, 30 µm. **(B)** FACS analysis of L1CAM expression (x-axis) in OVCAR3-scramble and OVCAR3-sh1 cells after transfection with an L1CAM construct for protein rescuing. Cells belonging to the different gates were then used for the experiments shown in Figure 1D. **(C)** Values of stem cell frequency calculated from the *in vitro* extreme limiting dilution experiment. OVCAR3-sh1 cells showed a lower stem cell frequency respect to the control scramble cells, thus reflecting a reduced OCSC content upon L1CAM silencing.

Supplementary Figure S3. L1CAM ectopic expression in Ov90 OC cell line. (A) Representative immunofluorescence images of Ov90-mock and Ov90-L1CAM stained for L1CAM (red) and counterstained with DAPI (blue). Arrows indicate L1CAM membrane localization. Scale bars, 10 µm. **(B)** qRT-PCR of bulk cells (black bars) and second-generation spheres (grey bars) from Ov90-L1CAM cells for the indicated stemness-related genes. Data is normalized to the three housekeeping genes HPRT1, ACTINB and GAPDH and is presented as a fold change respect to either bulk or OCSC mock control cells. L1CAM ectopic expression significantly increased POU5F1 (OCT4), CD44, and TWIST1 while decreasing CD24 mRNA level. Data are expressed as means ± SD from a representative experiment performed in triplicate. Comparisons between experimental groups were done with two-sided Student's t-tests; **$p<0.01$, ***$p<0.001$. **(C,D)** Values of stem cell frequency calculated from the *in vitro* (C) and *in vivo* (D) extreme limiting dilution experiment. Ov90-L1CAM cells exhibited a higher stem cell frequency respect to the control cells, consistent with an increased OCSC content upon L1CAM ectopic expression.

Supplementary Figure S4. L1CAM expression in OC primary samples. (A) FACS analysis for L1CAM expression (x-axis) on three independent OC primary samples. Cells belonging to gate P5 (orange) were sorted out as L1CAM-positive while those belonging to gate P6 (green) were sorted as L1CAM-negative. This experiment refers to the OC samples shown in Figure 1H.

Supplementary Figure S5. L1CAM silencing in SKOV-3 cells. (A) Immunoblotting for L1CAM on L1CAM-knockdown SKOV-3 cells showing L1CAM silencing. These cells were used in the *in vivo* experiment shown in Figure 2A. Vinculin served as loading control.

Supplementary Figure S6. Chemotherapy response of L1CAM-transduced adherent cells. (A) Ov90-mock and Ov90-L1CAM cultured as monolayers were treated for 5 days with increasing concentrations of paclitaxel in quadruplicate. Following treatment, cell viability was measured with WST-8 kit and IC_{50} was calculated using GraphPad Prism 8. IC_{50} values are indicated. L1CAM did not impact the response of Ov90 cells to paclitaxel under adherent conditions. **(B,C)** Sphere formation assays were performed on OVCAR3 (B) or primary OC cells (C) in the presence of 20 μg/ml CE7 alone, 2.5 μg/ml carboplatin (carbo) alone, or their combination. ***p<0.001; *p<0.05.

Supplementary Figure S7. L1CAM-related signaling in OCSC. (A) Gene Set Enrichment Analysis (GSEA) of Ov90-OCSC of STAT3 pathway (BioCarta) found significantly enriched in L1CAM overexpressing OCSC vs. mock treated OCSC. **(B)** Immunoblotting for both phospho-STAT3 (pSTAT3) and total STAT3 (STAT3) showing that L1CAM silencing in OVCAR3 second-generation spheres decreased STAT3 activation. Actin served as loading control. **(C)** Immunofluorescence of serum-starved Ov90-mock and Ov90-L1CAM for phospho-STAT3 (pSTAT3; red,) and total STAT3 (STAT3; cyan) counterstained with DAPI (blue). Arrows in the merged staining (fourth panel) indicate pSTAT3-positive nuclei which were more frequent in L1CAM-expressing cells. Quantification of immunofluorescence is provided in the graph. Data are expressed as the percentage of pSTAT3-positive over the total of STAT3-positive nuclei in five different microscope fields, and are shown as means ± SEM. Scale bars, 20 μm. Comparisons between experimental groups were done with two-sided Student's t-tests; **p<0.01. **(D)** Representative image of an OC sample stained for L1CAM (red) and pSTAT3 (brown). Arrows show the co-localization of the two proteins. **(E)** Immunoblotting for both phospho-SRC (pSRC) and total SRC (SRC) showing that L1CAM silencing in OVCAR3 second-generation spheres decreased SRC activation (phosphorylation). Actin served as loading control.

Supplementary Figure S8. Apoptosis induced by STAT3 inhibition and chemotherapy in L1CAM-expressing OC cells. Ov90-mock and Ov90-L1CAM cells were treated with 6nM Paclitaxel alone, 250nM Napabucasin alone, or their combination, for 24 hours, followed by apoptosis assay with Caspase-Glo 3/7. Caspase 3/7 activity was normalized on the number of cells. Data are expressed as the ratio between drug-treated cells and control cells treated with the vehicle only. *p<0.05; **p<0.01; ***p<0.005.

Supplementary Tables

Supplementary Table S1. Differentially expressed genes in the bulk (adherent) populations of Ov90-mock vs Ov90-L1CAM cells.

Supplementary Table S2. Differentially expressed genes in the OCSC (sphere-derived) populations of Ov90-mock vs Ov90-L1CAM cells.

Supplementary Table S3. Top Canonical Pathway enriched in L1CAM-regulated genes.

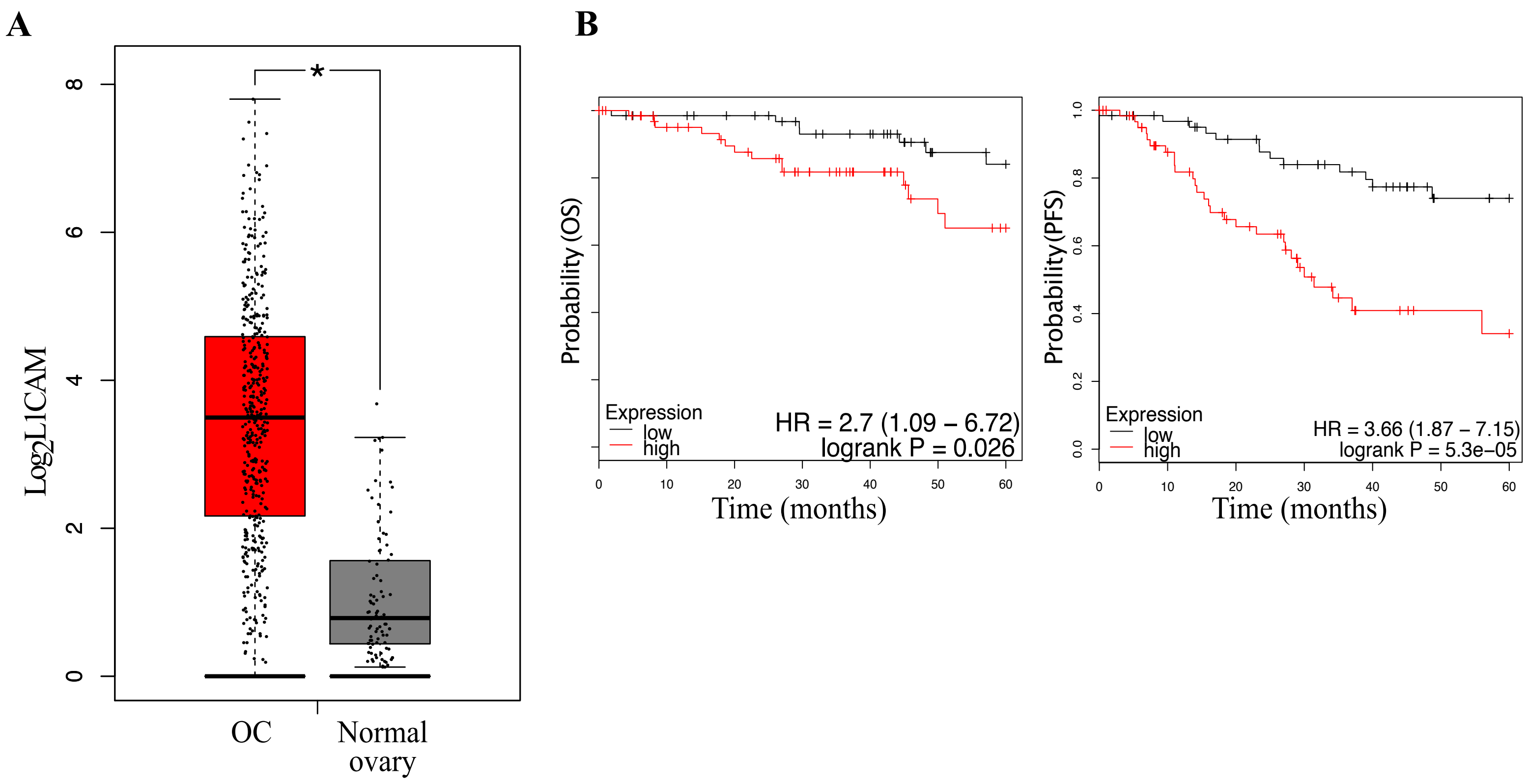

Supplementary Fig. S1

A

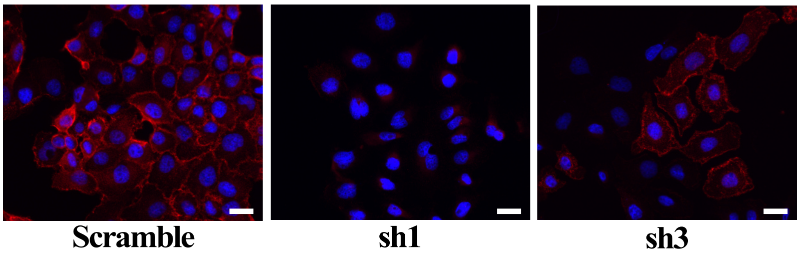

B

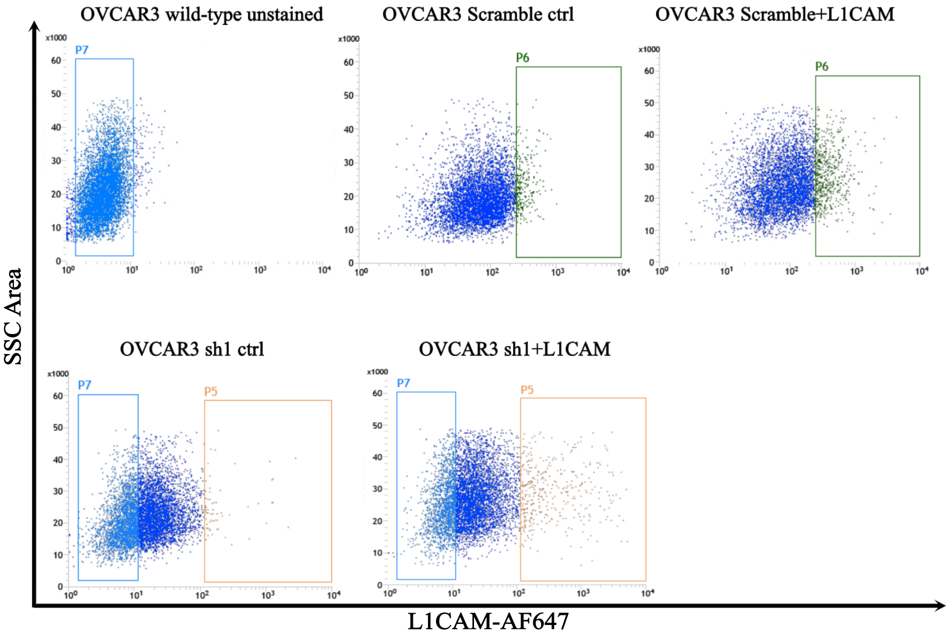

C

Group	Stem cells frequency	p-value
Scramble	1/19	<0.001
sh1	1/147	

Supplementary Fig. S2

A

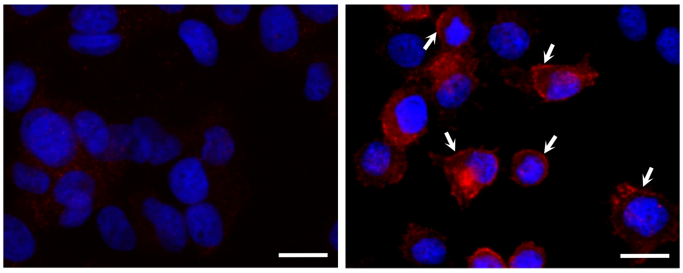

Ov90-mock **Ov90-L1CAM**

B

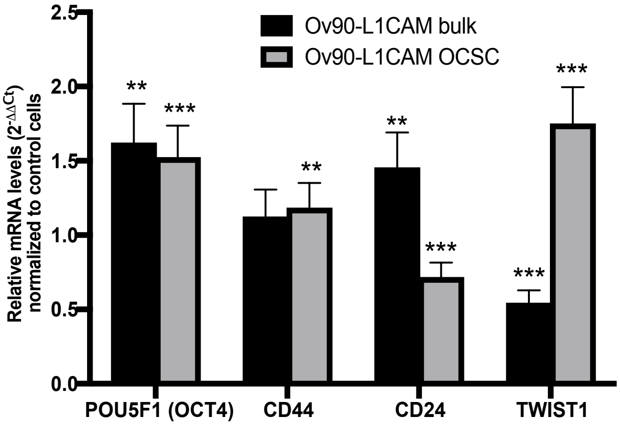

C

Group	Stem cells frequency	p-value
Ov90-mock	1/200	0.02
Ov90-L1CAM	1/64	

D

Group	Stem cells frequency	p-value
Ov90-mock	1/5263	0.007
Ov90-L1CAM	1/20000	

Supplementary Fig. S3

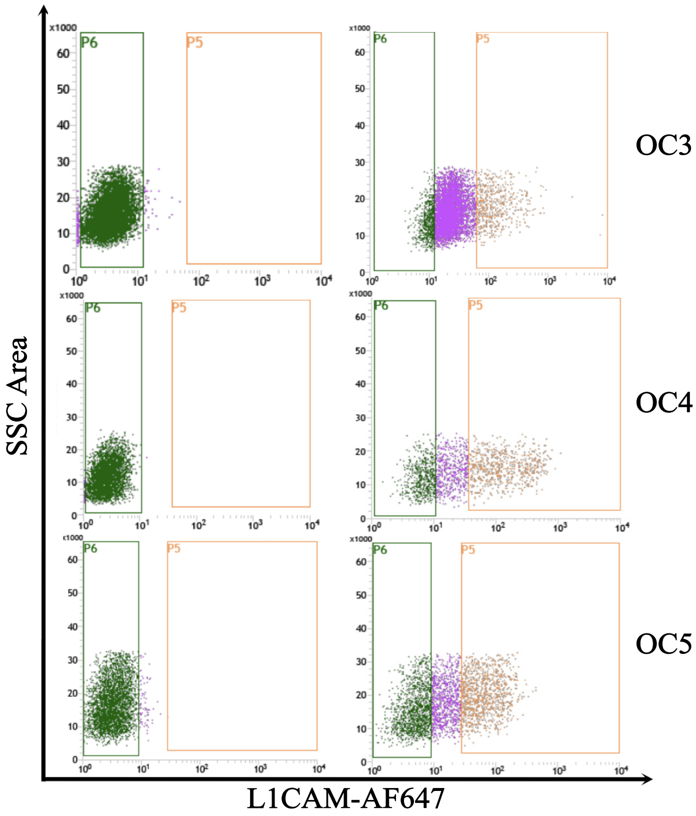

Supplementary Fig. S4

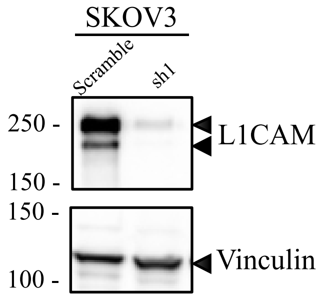

Supplementary Fig. S5

A

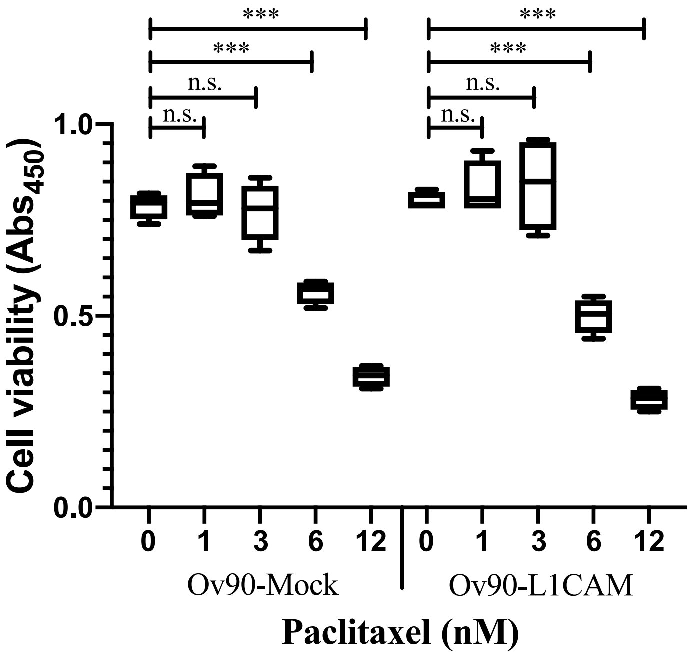

B

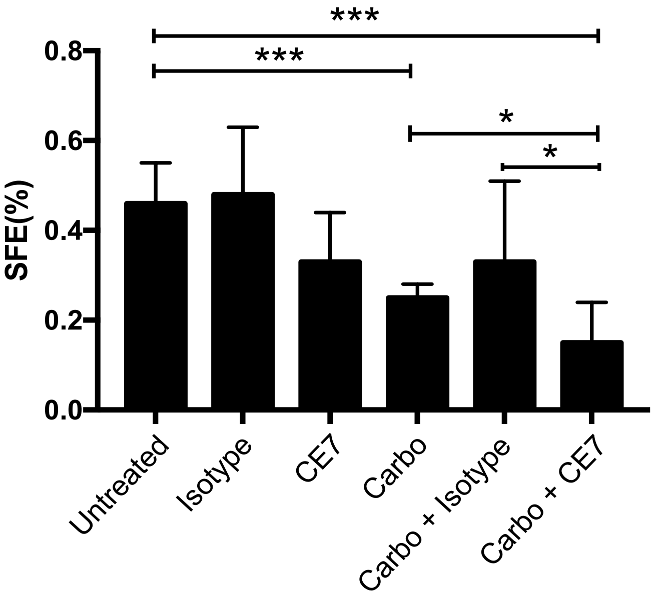

C

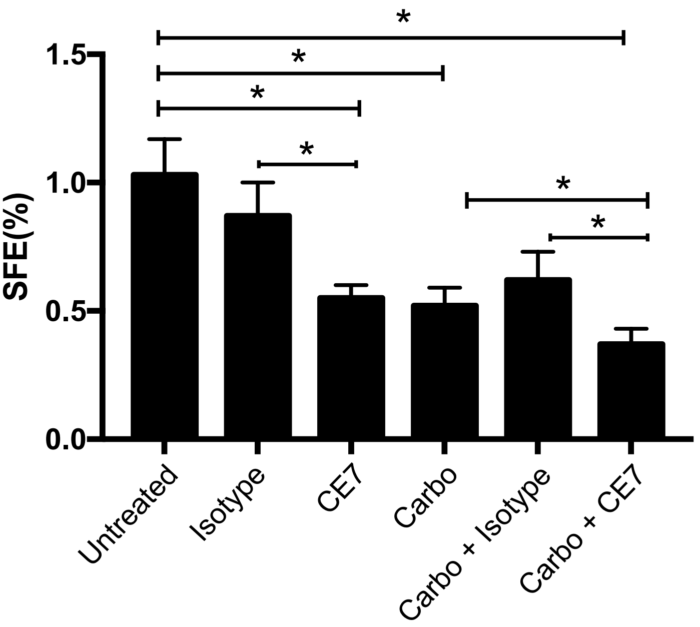

Supplementary Fig. S6

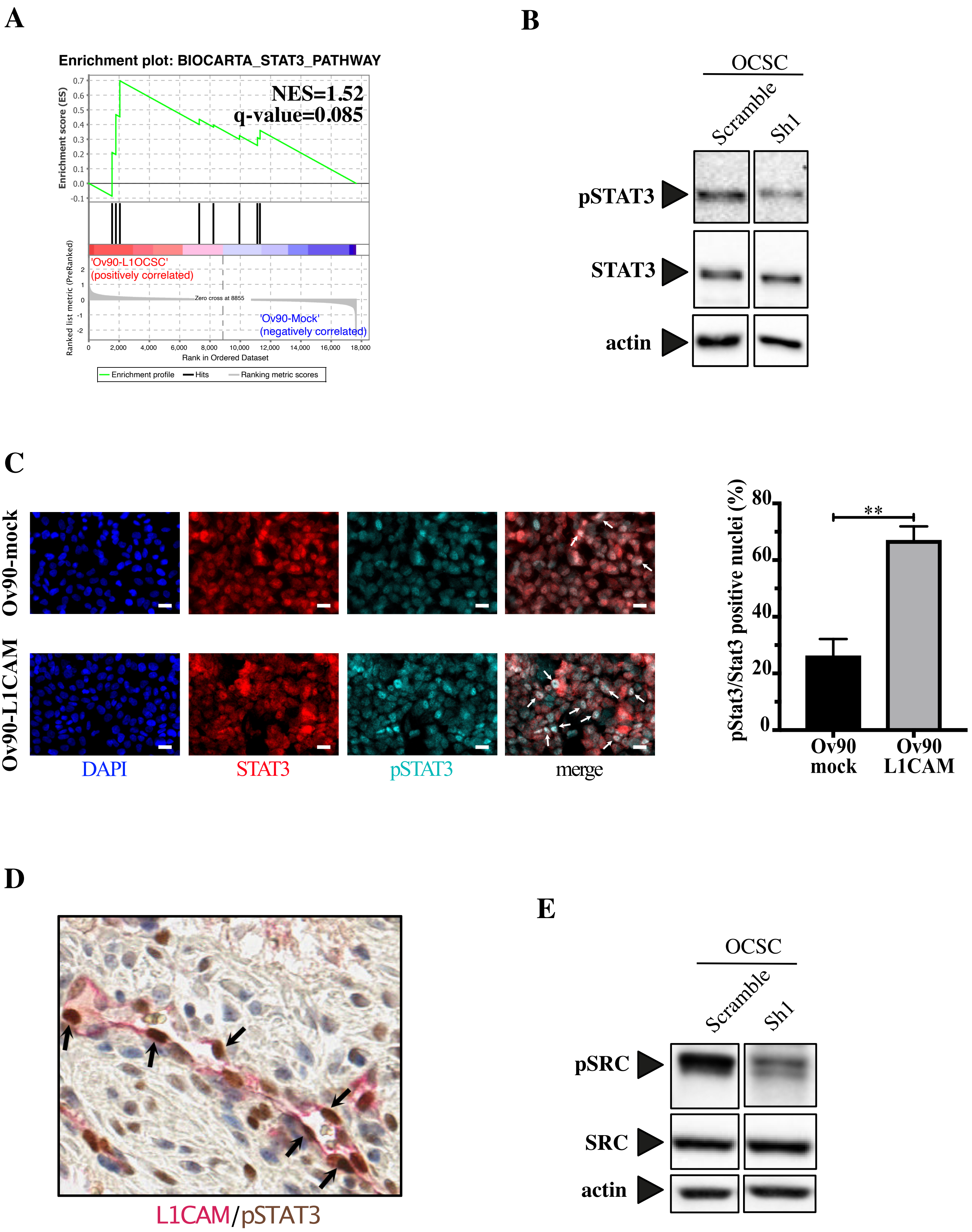

Supplementary Fig. S7

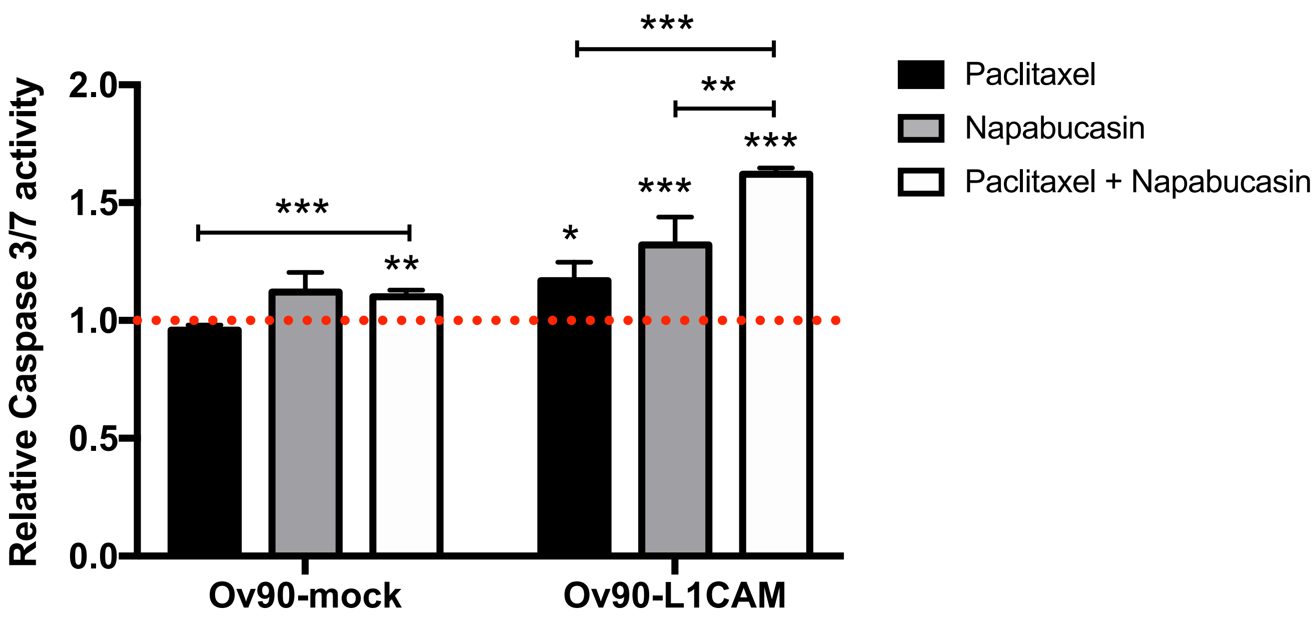

Supplementary Fig. S8
